# Navigating from live to virtual social interactions: looking at but not manipulating smartphones provokes a spontaneous mimicry response in the observers

**DOI:** 10.1007/s10164-021-00701-6

**Published:** 2021-04-17

**Authors:** Veronica Maglieri, Marco Germain Riccobono, Dimitri Giunchi, Elisabetta Palagi

**Affiliations:** Unit of Ethology, Department of Biology, University of Pisa, Via A. Volta 4, 56126 Pisa, Italy

**Keywords:** Human ethology, Nonverbal communication, Use of devices, Objective social isolation, Perceived social isolation

## Abstract

By gathering data on people during their ordinary daily activities, we tested if looking at, but not manipulating, smartphones led to a mimicry response in the observer. Manipulating and looking at the device (experimental condition), more than its mere manipulation (control condition), was critical to elicit a mimicry response in the observer. Sex, age and relationship quality between the experimenter and the observer had no effect on the smartphone mimicry response that tended to decrease during social meals. Due to the role of food as a tool in increasing social affiliation, it is possible that during communal eating, people engage in other forms of mimicry involving facial expressions and postures rather than the use of objects. Understanding the ethological mechanisms of the use of smartphones at everyday-social scale could unveil the processes at the basis of the widespread/increasing use of these devices at a large scale.

**Supplementary Information:**

The online version contains supplementary material available at 10.1007/s10164-021-00701-6.

## Introduction

The use of smartphones is defined as “*any application of the cell phone as a tool, including talking, text messaging, game playing or the sheer accessibility of the instrument*” (Banjo et al. 2008, p. 127). In 2020, the diffusion of smartphones reached the 44.81% of the population all over the world with 3.50 billion of users (Statista 2020). Smartphones are regularly used by people, from the youngest to the oldest ones, not only to call but also for texting, surfing on internet, playing or taking pictures and videos (Banjo et al. 2008). Smartphones are used in a wide variety of social situations such as places of entertainment (cinemas, theatres), parks, public transportation (bus, train), waiting rooms, restaurants and job spaces (Park 2019; Manzerolle 2013). On the one side, smartphones have the function to socially connect people who are spatially distant (Manzerolle 2013), thereby favour the sharing of information at a large scale (Oulasvirta et al. 2012). On the other side, the use of smartphones can increase social isolation through interference and disruption with real-life ongoing activities (Bugeja 2005; Gill et al. 2012). This may cause distress (Chesley 2005), and in some cases, reduce the sense of volitional control (Thomée et al. 2011). The use of smartphones in public spaces is transforming our roles from active social participants to inactive observers, thus making us mere bystanders of our social lives (Cumiskey 2005). While using smartphones, people are less likely to give help to others and engage in less nonverbal social behaviours, such as smiling, that are crucial to the communicative exchange during social interactions (Banjo et al. 2008). Finally, the negative consequences generated by a compulsive use of smartphones can lead to symptoms, usually described as “dependence” or “addiction” (Chen et al. 2017), that can have repercussions also on entire societies (Park 2019). Young women seem more subjected to this kind of addiction. In fact, Lee and co-workers (2016) demonstrated that in South Korea young women were significantly more dependent on smartphones than young men. This dependence translates into negative feelings such as anxiety and sense of insecurity in case of smartphone deprivation (Lee et al. 2016).

Understanding the ethological mechanisms at the basis of the use of these devices could help explain if and how the different social contexts affect the individual motivation to make use of smartphones. The unintentional, unconscious imitation is one of the mechanisms at the basis of the diffusion of some behavioural traits (Chartrand and Lakin 2013; van Baren et al. 2009). Humans often mimic spontaneously others’ behaviours, without being aware of doing it (Chartrand and Lakin 2013; Van Baaren and Fockenberg 2006). Mimicry occurs when two or more individuals unintentionally engage in the same behaviour at (more or less) the same time (Chartrand and Lakin 2013). To describe this phenomenon, some scholars coined the term “chameleon effect”, which refers to the passive and unintentional behavioural matching between two or more subjects in a given social situation (Lakin and Chartrand 2003; Chartrand and Bargh 1999).

In humans, mimicry can involve expressions, gestures, postures and other motor movements that vary from yawning to foot shaking and vocal accent (Palagi et al. 2020; Genschow et al. 2017; Chartrand and Lakin 2013; Herrmann et al. 2011; Tiedens and Fragale 2003; Giles et al. 1991; La France 1982) and can even involve objects such as pens or cigarettes (Harakeh and Vollebergh 2012; Stel et al. 2010; Harakeh et al. 2007; van Baaren and Fockenberg 2006).

Mimicry plays a role in forming new social relationships as well as nourishing already established social bonds (Chartrand and Lakin 2013). Subjects that have recently experienced social exclusion tend to mimic their valuable partners more than subjects who did not suffer the same negative experience (Lakin and Chartrand 2005, 2012; Over and Carpenter 2009; Lakin et al. 2008). People mimic ingroup members, such as kin and friends, more than outgroup members such as strangers (Palagi et al. 2020; Bourgeois and Hess 2008; Likowski et al. 2008; McIntosh 2006; Tickle-Degnen 2006; Yabar et al. 2006).

One of the contexts in which social bonds can be created and strengthened is communal eating. During social meals, people feel happier and increase trusting of others by sharing not only food but also postures, facial expressions and experiences (Dunbar 2017). Eating alone can cause stress and depression thus increasing the perceived social isolation (Kim et al. 2020).

To test if the “chameleon effect” is one of the possible determinants of the widespread use of smartphones in social contexts, we gathered data on people that were unaware to be observed in their naturalistic social settings. After the administration of an experimental and a control stimulus, we evaluated the presence and latency of spontaneous mimicry response in the observers. In the experimental condition, the experimenter took, kept in hands and manipulated his/her smartphone (fiddling and swiping) while *looking at the screen* for at least 5 s. In the control condition, the experimenter took, kept in hands and manipulated his/her smartphone (fiddling and swiping) for at least 5 s *without looking at the screen*. This approach allows understanding whether the attention that the trigger devotes to the smartphone, more than its mere manipulation, provokes a congruent mimicry response in the observer.

According to the data indicating that young people, and particularly women, make a large use of smartphones during their social interactions (Lee et al. 2016; Srivastava 2005; Campbell 2005), we expect that young subjects, especially women, are more infected by seeing others using smartphones. If mimicry in the use of objects, as it occurs for facial and bodily mimicry (Palagi et al. 2020; Stel et al. 2010; Likowski et al. 2008; Bourgeois and Hess 2008; McIntosh 2006; Tickle-Degnen 2006; Yabar et al. 2006), is predictive of social bonding, we expect that the mimicry in the use of smartphones follows a positive gradient of familiarity from strangers to kin. Lastly, if communal eating has a role in maintaining people under live social sphere by reducing their urge to mimic others in navigating in virtual interactions, we expect that people show lower mimicry response in the use of smartphones under feeding contexts.

## Materials and methods

### Ethic statement

The present study has been authorized by the Committee on Bioethics of the University of Pisa (Review No. 5/2020; AOO “CLE”—Prot.: 0036356/2020 of 10/04/2020). The study was purely observational and data were entered in an anonymous form (an alphanumerical code has been uniquely assigned to each subject). People have been observed in their natural social setting without any modification of their ordinary and daily activities.

### Data collection and subjects

The data were collected in Italy across 5 months (May–September 2020) compatibly with the *d.l. n.33* “*further urgent measures to contrast the epidemiological emergency from COVID-19*” issued by the Italian Government on May 16th, 2020.

The observations were temporally distributed across morning (from 07:00 am to 01:00 pm), afternoon (from 01:00 pm to 07:00 pm) and night (from 07:00 pm to 03:00 am). Experimenters observed subjects in their natural social settings during their daily activities (at work, restaurants, cinemas, gyms, waiting rooms, social parties, social meals, public parks, family environments, etc.). The subjects, who were unaware to be observed (blind data collection), were people known (family members, friends, acquaintances and co-workers) and unknown (strangers) to the experimenters. The observed persons could know each other or not. A total of 184 persons (88 women, 96 men) were observed and included in the dataset.

To be included in the analysis, the sequence of actions had to fulfil several criteria both during the Experimental (EC) and Control condition (CC). The CC had to be identical to the EC except for the presence of the behaviour “*looking at the screen*”. During the EC, we considered as trigger the person who took, kept in hands, and manipulated his/her smartphone (e.g. fiddling and swiping) and *looked at the screen* for at least 5 s. During the CC, we considered as trigger the person who took, kept in hands, and manipulated his/her smartphone (e.g. fiddling and swiping) for at least 5 s *without looking at the screen*. In both conditions, the screen of the device had to be visible and not covered by any cover. Only those events in which the device automatically illuminated by touching were included in the dataset, since the light had to be present both in EC and CC. The two different conditions were randomly distributed, and the different observation bouts were separated by at least 10 min. The main triggers were M.G.R (male) and V.M. (female) who were the experimenters as well. We opportunistically gathered data also when other people (unconscious male and female triggers), not aware of the ongoing study, spontaneously manipulated/looked at their own smartphones for at least 5 sec.

The observer was defined as the person who visually perceived the triggers’ action and had his/her smartphone within reach. In short, the observer should have the opportunity to engage in the same action of the trigger in both EC and CC. The experimenters had to be able to see the gaze of the observers during both EC and CC. Immediately after the trigger took the device (*t*_0_), all individuals visually perceiving the triggers’ action were observed for 3 min by the experimenters who checked for the presence/absence of a congruent mimicry response in the observers.

The latency in the response (when present) was scored on six levels made of 30-s blocks with the aid of a wristwatch (Casio F-91W-1YER-P), a device that allows checking time without any kind of manipulation and light production (no illuminated screen).

Before starting systematic data collection, reliability between the experimenters (V.M.; M.G.R.) was tested. During 15 observational sessions, both experimenters gathered data concurrently on the same observers. At the end of the training period, the Cohen’s kappa values (*k*) were calculated for (i) the opportunity to be seen by the potential observers, (ii) the occurrence of the mimicry event, and (iii) the time latency. For all these conditions, the *k* values were always higher than 0.85 (Kaufmann and Rosenthal 2009). At regular intervals, to check for reliability, both experimenters collected data concurrently on the same group of people always obtaining k values higher than 0.85.

After 3 min, to make the data registration possible and unnoticed, the experimenters moved away from the observed subjects and took notes of their behaviour on smartphones or paper. The identity of the observed subjects was stored under alphanumerical codes.

We excluded from the database all the cases in which people, while using their smartphone, actively solicited the observers’ attention by indicating/showing the device (nonverbal solicitation) and/or verbally inviting to use it (e.g. “*look at that video*” and “*look at that post on…*”).

### Operational definitions

Both in EC and CC, we recorded the behaviour of the observer (presence of mimicry response/absence of mimicry response) during a 3-min time slot after seeing the trigger’s action. The occurrence of mimicking was coded as 1 (presence) or 0 (absence).

The response latency was measured as the time delay between the first touching of the smartphone by the trigger (*t*_0_) and the first touching of the smartphone by the observer (*t*_x_). The time latency was scored on six levels: 0 < *t*_x_ ≤ 30 s = 1; 30 s < *t*_x_ ≤ 1 min = 2; 1 min < *t*_x_ ≤ 1.5 min = 3; 1.5 min < *t*_x_ ≤ 2 min = 4; 2 min < *t*_x_ ≤ 2.5 min = 5; 2.5 min < *t*_x_ ≤ 3 min = 6.

The timing of observations was clustered as follows: morning (07:00 am–01:00 pm) = 0; afternoon (01:00 pm–07:00 pm) = 1; night (07:00 pm–03:00 am) = 2.

We recorded and categorized the sex (men = 0; women = 1) and age of the trigger and observer (18–25 years = 0; 26–40 years = 1; 41–60 years = 2).

The relationship between the trigger and the observer was clustered on four categories: strangers (people who had never met before = 0), acquaintances (people who exclusively shared an indirect relationship based on a third external factor—work duty, colleagues, friends in common, friends-of-friends = 1), friends (not kin subjects sharing a direct friendship relationship = 2), regular engaged partners and kin (family members and cohabitants = 3). In most cases, the relationship between the observed people was known to the experimenters. When the trigger was different from and unknown to M.G.R. and V.M., the experimenters collected personal information (e.g. age, relationship between the observed subjects) by engaging in a friendly conversation. When it was not possible to gather information on the age of the observed subjects or on their relationship, we excluded the record from the dataset.

Since food is a strong factor of affiliation in humans, we also categorized the social context in which the data have been recorded as a function of the absence = 0 or presence of food = 1. The context “presence of food” began when the subjects sat down at the table and ended when they left the table. In addition, during meals, subjects had the opportunity to manipulate their devices if they wanted. Social breakfasts, lunches, dinners and happy hours were included in the cluster ‘presence of food’. All the other social contexts such as working, travelling, relaxing time, board gaming, card gaming, studying in libraries and waiting in a sitting room (e.g. hair dressing salons, dentist studios) were clustered as ‘absence of food’.

Experimental and control conditions were randomly distributed across all the possible contexts and the periods of the day.

### Data analysis and statistics

From a total of 820 events (N_EC_ = 472; N_CC_ = 348) involving 184 subjects (women = 88; men = 96), we extracted for the analysis 721 events (*N*_EC_ = 386; *N*_CC_ = 335) involving 103 subjects (women = 50; men = 53) that were tested for both conditions (EC and CC). To investigate the factors affecting the mimicry response in the use of smartphones, we ran a Generalized Linear Mixed Model (GLMM) with a binomial error distribution by means of the R-package *glmmTMB* 1.2.5042 package (Brooks et al. 2017), using absence/presence of mimicry as response variable. We included only the subjects who had at least one observation in the EC and one in CC (*N* = 721 cases). The fixed effects were the condition (Control condition, CC; Experimental condition, EC), the age of the trigger and the receiver (18–25 years; 26–40 years; 41–60 years), the sex of the trigger and the receiver, the level of familiarity between the trigger and the receiver (strangers; acquaintances; friends; kin), the period of the day (morning, afternoon and night), and the context (presence of food; absence of food). The identities of the trigger and the receiver were entered as random factors.

The overall significance of the full model was tested by comparing this model with the model including only the random effects (Forstmeier and Schielzeth 2011) by means of the Likelihood Ratio Test (LRT; Dobson 2002). The LR test was used also to test the significance of the fixed factors using the function *Anova* in the R-package *car* 3.0–10 (Fox and Weisberg 2019). To exclude the occurrence of collinearity among predictors, we examined the variance inflation factors (VIF; Fox 2016) by means of the R-package *performance* 0.4.4 (Lüdecke et al. 2020). No collinearity has been found between the fixed factors (range VIF_min_ = 1.06; VIF_max_ = 1.57). Model fit and overdispersion were checked using the R-package *DHARMa* 0.3.3.0 (Hartig 2020). The marginal R^2^, which represents the variance explained by fixed factors only, and the conditional *R*^2^, which represents the variance explained by the entire model including both fixed and random effects (Nakagawa et al. 2017), were calculated using the R-package *MuMIn* 1.43.17 (Bartoń, 2020). Then, we used the “confint(x)” function to interpret the estimated effects as relative odds ratios. Relative odds ratio (i.e. the expected odds change for one unit increase in the explanatory variable when the remaining variables are set to their reference category) were used to evaluate the magnitude of the estimated effects. We performed all pairwise comparisons for the levels of the multilevel factor with the Tukey test (Bretz et al. 2010) using the R package *emmeans* (Length et al. 2020).

Lastly, to test whether the distribution of the mimicry response was homogenous across the six 30-s time windows, we applied the Chi-square test. From the original dataset (*N* = 820 events), we included in this analysis only the mimicry events occurred in the six different 30-s time window slots during the EC (*N* = 249 cases; women = 54, men = 59). All calculations were performed using R 4.0.3 (R Core Team 2020).

## Results

To investigate the presence of mimicry in the use of smartphone, we ran a GLMM with a binomial error distribution using the absence/presence of mimicry as response variable. The fixed effects were: the condition (Control condition, CC; Experimental condition, EC), the age class of the trigger and the observer (18–25 years; 26–40 years; 41–60 years), the sex of the trigger and the observer, the level of familiarity between the trigger and the observer (strangers; acquaintances; friends; kin), the period of the day (morning, afternoon and night) and the context (presence of food; absence of food). The ID of the trigger and the observer were included as random factors.

The full model, including all the fixed factors, was significantly different from the null model, comprising only the random factors (likelihood ratio test: *χ*^2^ = 186.5, df = 8, *p* < 0.001; marginal *R*^2^ = 0.473; marginal *R*^2^_full model_ − marginal *R*^2^_null model_ = 0.425; conditional *R*^2^ = 0.537; conditional *R*^2^_full model_ − conditional *R*^2^_null model_ = 0.482).

The fixed factors ‘condition’, ‘period of the day’ and ‘context’ had a significant effect on the mimicry response (Table 1). The likelihood of the occurrence of the mimicry response was about 28 times (odds ratio = 28.197) higher in the experimental (EC) compared to the control condition (CC) thus indicating that people were more susceptible to use smartphones when the triggers focussed their attention on the screen of their devices compared to when they were simply manipulating them.

**Table 1:** Estimated parameters (Coeff), standard error (SE), 95% confidence intervals (2.5–97.5% CI), and results of the likelihood ratio tests ($\chi^{2}$) of the Generalized Linear Mixed Model (with a binomial error distribution) investigating the effect of the condition (experimental condition vs control condition), the period of the day (morning, afternoon, night), context (presence/absence of food), the sex of the trigger and the observer, the age of the trigger and the observer (18–25 years; 26–40 years; 41–60 years), and the level of familiarity (strangers; acquaintances; friends; kin) between the trigger and the observer on the presence/absence of mimicry

Fixed effects	Coeff	SE	2.5% CI	97.5% CI	$\chi^{2}$	df	*P*
Intercept	-2.514	0.656	− 3.801	− 1.227			
Condition	3.339	0.328	2.697	3.982	103.794	1	**0.000**
Period of the day					6.025	2	**0.049**
Period [afternoon]	− 0.696	0.363	− 1.407	0.014			
Period [night]	− 0.892	0.364	− 1.605	− 0.178			
Context	− 0.612	0.248	− 1.105	− 0.134	0.013	1	**0.013**
SEX_trigger_	0.262	0.278	− 0.282	0.806	0.894	1	0.344
SEX_observer_	0.113	0.287	− 0.449	0.675	0.156	1	0.693
AGE_trigger_					3.445	2	0.179
AGE_trigger_ [26–40 yrs]	0.007	0.370	− 0.718	0.731			
AGE_trigger_ [41–60 yrs]	1.017	0.631	− 0.220	2.255			
AGE_observer_					4.732	2	0.094
AGE_observer_ [26–40 yrs]	− 0.211	0.327	− 0.852	0.430			
AGE_observer_ [41–60 yrs]	− 0.942	0.442	− 1.808	− 0.077			
Level of familiarity					5.015	3	0.171
Familiarity [acquaintances]	− 0.078	0.478	− 1.014	0.858			
Familiarity [friends]	− 0.118	0.461	− 1.021	0.785			
Familiarity [kin]	0.684	0.485	− 0.267	1.635			

*N*_cases_ = 721, *N*_exp_ = 386; *N*_control_ = 335; *N*_observers_ = 104, *N*_triggers_ = 44. Variance for the random factors: ID_trigger_ = 2.57e^−9^, SD = 5.07e^−5^, ID_observers_ = 0.457, SD = 0.676

The significant values are indicated in bold

As for the period of the day, the mimicry response tended to be more frequent in the morning compared to the night period (Tukey test: *t*-ratio = 2.448; df = 705; *p* = 0.039), whereas no difference was found in the response either between the morning and the afternoon (Tukey test: *t*-ratio = 1.921; df = 705; *p* = 0.134) or between the afternoon and the night (Tukey test: *t*-ratio = 0.738; df = 705; *p* = 0.741). Finally, the mimicry response was less likely in presence of food (Table 1).

The time latency of the mimicry response was highly dishomogeneous across the six 30-s time windows considered (Chi-square test, *χ*^2^ = 24,911.71; df = 5; *p* < 0.0001) with almost all the response events occurring in the first 30-s time window (*N*_0<*t*x≤30 sec_ = 212; *N*_30sec<*t*x≤1 min_ = 15; *N*_1<*t*x≤1.5 min_ = 3; *N*_1.5<*t*x≤2 min_ = 4; *N*_2<*t*x≤2.5 min_ = 2; *N*_2.5<*t*x≤3 min_ = 0).

## Discussion

**Fig. 1:**
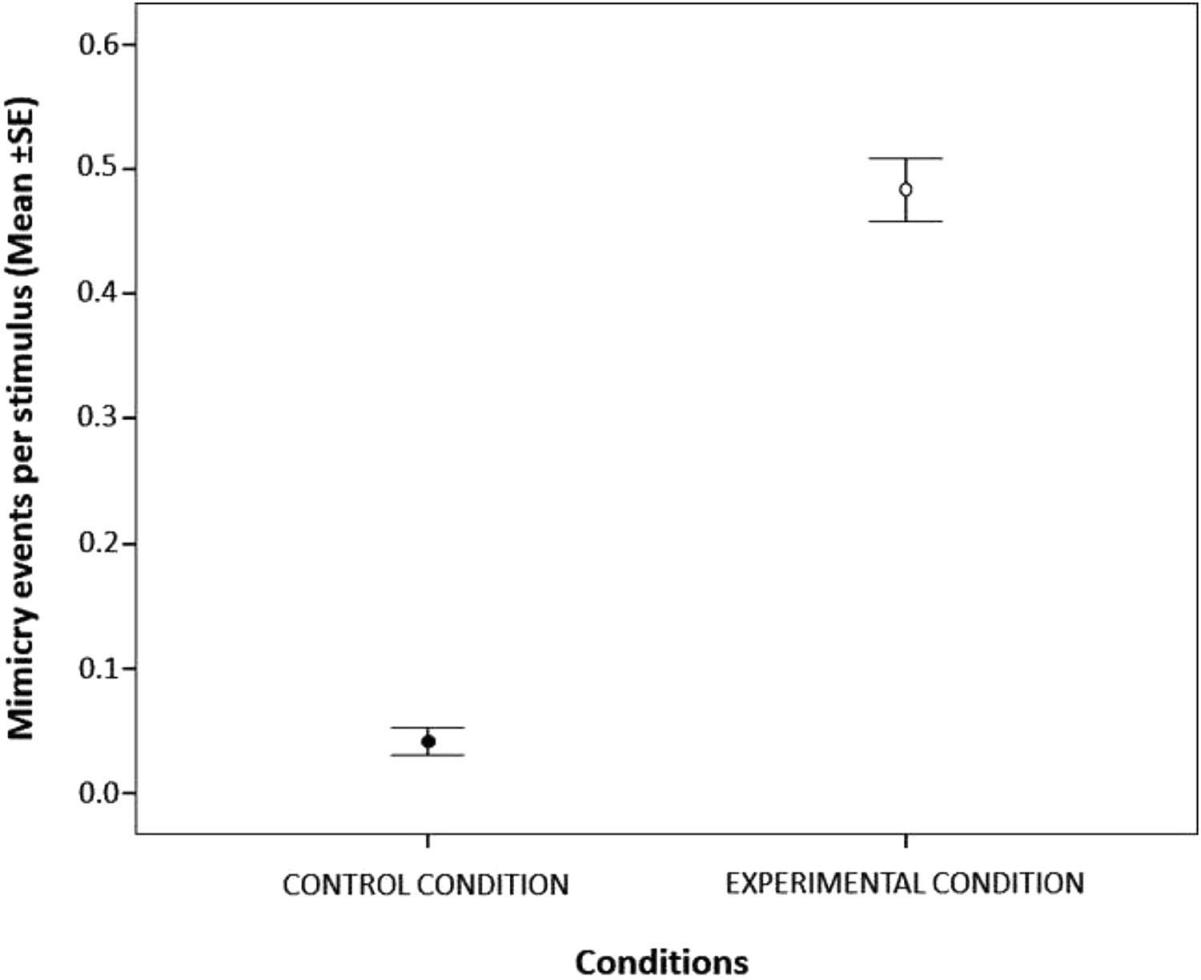
Mean ± standard error (SE) of the number of the mimicry response events per stimulus in the two different conditions (experimental, open dot; control condition, black dot)

Here, we showed that manipulating a smartphone is not sufficient per se to elicit a mimicry response in the observers. In fact, mimicry becomes evident only when the trigger is looking at the device (Fig. 1). The short time latency (< 30 s) of the response indicates a certain level of automaticity and spontaneousness of the phenomenon (van Baren et al. 2009). In the literature, there is a debate about the degree to which mimicry phenomena are goal- or movement-based. While some scholars argue that observers extract the high-level goal of an observed behaviour and use this goal as a guide for their own course of action (e.g. Wohlschläger et al. 2003; Bekkering et al. 2000), others suggest that individuals merely match specific movements to the observed ones without necessarily sharing or adopting the same goal (e.g. Genschow et al. 2013). Since the mimicry effect was evident only when the triggers devoted their own attention to the device, our finding seems to support the goal-directed hypothesis. The main goal (here the attention to the device and not its simple manipulation) can activate in the observer the motor programme that is mostly associated with the achievement of that goal (Wohlschläger et al. 2003). However, further data on the mimicry in the use of smartphones will be necessary to fully understand the proximate factors at the basis of the behaviour.

**Fig. 2:**
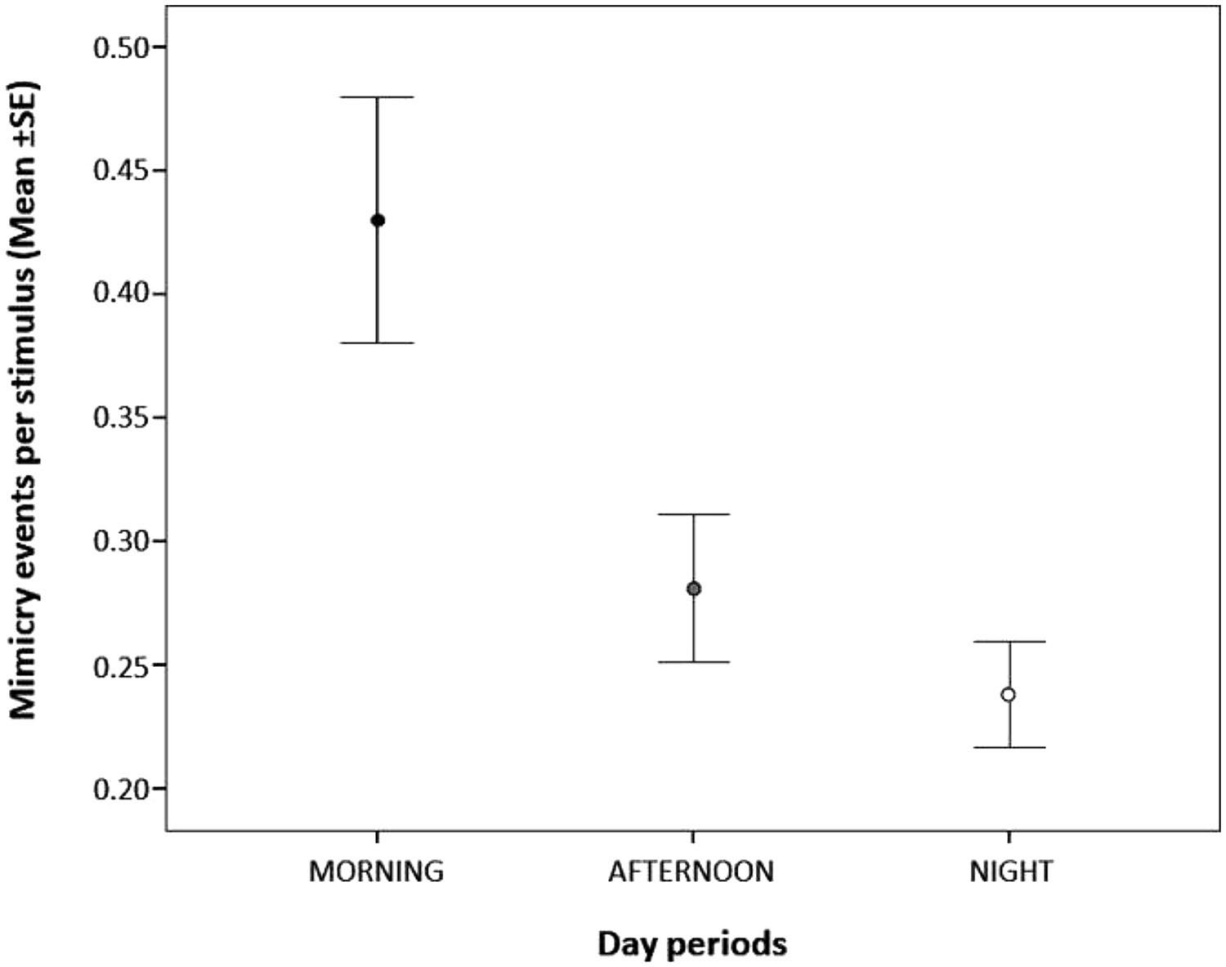
Mean ± standard error (SE) of the number of the mimicry response events per stimulus events across the three periods of the day (morning, black dot; afternoon, grey dot; night, open dot)

**Fig. 3:**
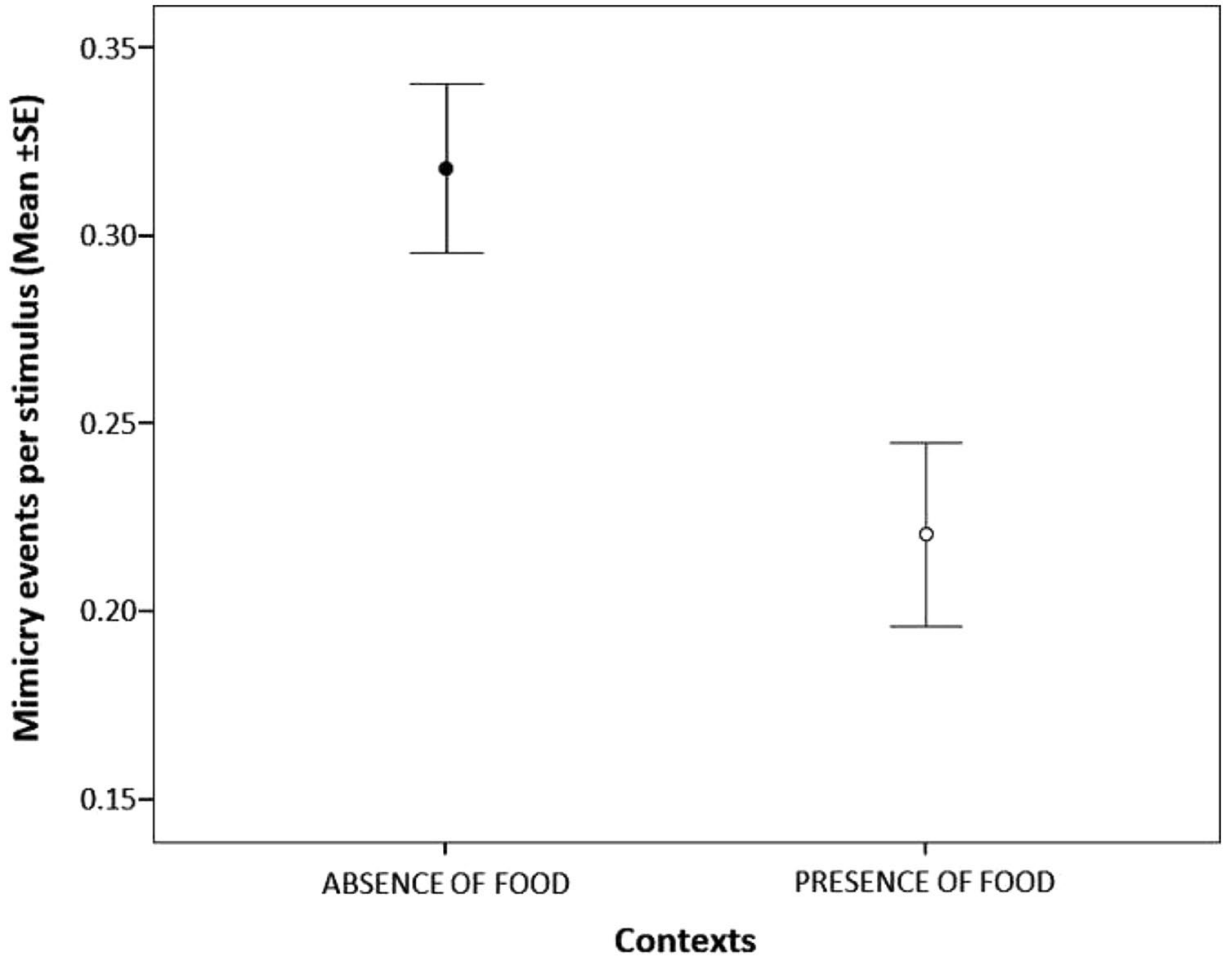
Mean ± standard error (SE) of the number of the mimicry response per stimuli events in the two different contexts (presence of food, open dot; absence of food, black dot)

Contrary to other kinds of mimicry (Palagi et al. 2020; Stel et al. 2010; Likowski et al. 2008; Bourgeois and Hess 2008; McIntosh 2006; Tickle-Degnen 2006; Yabar et al. 2006), we did not find any effect of age, sex and level of familiarity on the occurrence of the mimicry response, thus suggesting that mimicry in the use of smartphones is not liable to either individual (sex, age) or social preferences (familiarity). The period of the day had a significant effect on the occurrence of mimicry that peaked from 07.00 am to 01.00 pm (Fig. 2). The social events linked to the presence of food such as breakfasts, brunches, lunches, happy hours and dinners were characterized by the lowest levels of mimicry response as hypothesized (Fig. 3).

Working at everyday-social scale, unconscious mimicry in the use of smartphones can have repercussions also at a large scale possibly being one of the mechanisms at the basis of the diffusion of these devices across societies. Obviously, mimicry can occur only when the observers own the device and have the possibility to handle it. People that do not possess a smartphone can suffer social exclusion due to the mimicry effect operating daily at a group level (Cacioppo et al. 2011). People, who refuse to use smartphones, can experience frustration or anger when their ingroup members are infected by others’ behaviour and begin to navigate from live to virtual social interactions. This can lead to two possible outcomes. From one side, people can reduce their social encounters with their ingroup members thus experiencing an *objective social isolation* (Hawkley et al. 2008; Ruiz 2007). On the other side, to avoid experiencing *perceived social isolation*, which is highly related to the quality of social bonding shared between ingroup members (Hawkley 2008), people can start using the device to conform to others.

The mimicry in the use of smartphones was higher in the morning compared to the night. The daily time course of mimicry in the use of smartphones differs from that recorded for other forms of mimicry such as contagious yawning that shows two daily peaks, in the morning and in the evening/night (Giganti and Zilli 2011). Since yawning can act as a synchronizer by aligning activities of a social group (Palagi et al. 2020), it has been argued that the evening/night peak could serve to communicate tiredness and the need for sleep. It is possible that the level of attention and alertness of people that in the morning generally peak can translate into higher level of susceptibility to undergo to smartphone mimicry. Such higher susceptibility could be also linked to a higher motivation to renovate virtual contacts after a period of short separation, that is the night. Moreover, people experiencing high levels of perceived social isolation also show peak levels of cortisol in the morning (Adam et al. 2006). It could be possible that during this period people can be more motivated to shorten social distance by mimicking others thus dealing with their negative emotional state. Due to the lack of studies on the mimicry response in the use of objects (e.g. cigarettes, pens) as a means to enter in contact with others and manage perceived social isolation, our hypothesis needs further investigations.

The low mimicry response recorded in presence of food suggests that people are more focussed on live social interactions during period of conviviality linked to the feeding contexts. Since data were collected before and after meals and between the courses, when people did not have their hands engaged in food consumption, our result does not suffer the limited opportunity of people to manipulate smartphones in presence of food. Food has both biological and social functions in human societies (Dunbar 2017). Beyond the biological functions (e.g. growth, health promotion, disease prevention), food can act as a “social cement” (Quandt 1999). Eating together and sharing food (e.g. happy hours, parties, working and everyday meals with colleagues, family or friends) provide both social and individual benefits across almost all human societies (Dunbar 2017). In this view, social meals are considered a “tool” for establishing and developing social bonds. The low mimicry response recorded in presence of food could be even more pronounced considering that our sample comes from Italy, where the culture of food is historically and intrinsically connected to social aggregation and conviviality (Helstosky 2005; Parasecoli 2004). It is possible that during communal eating, when people share memories and feel close to each other (Dunbar 2017), they can engage in other forms of mimicry involving facial expressions and postures (e.g. laughing together) rather that the use of objects. This hypothesis can also explain why the mimicry in the use of smartphones, contrary to other forms of facial and bodily mimicry (Palagi et al. 2020; Hess and Fischer 2013), is not affected by the level of familiarity between subjects. Using a smartphone does not communicate any information about the internal status of the user and does not probably have any function in the synchronization of the emotional state of interacting people.

Since the majority (89.83%) of the mimicry events occurred in the first 30-s time block, it is possible that our 30-s clustering could have hidden some possible effects of sex and age. Such effects could emerge if we would focus within the shortest time (30 s) window of the mimicry response. Now that there are indications on the presence of the mimicry phenomenon involving the use of smartphones, future studies should take into account what happens in this tight time window by measuring in a more precisely way the exact timing of the mimicry response (e.g. video collection).

Our findings further our understanding on mimicry in the use of smartphones at everyday-social scale and indicate that mimicry can be at the basis of the widespread use of these devices at a large scale. To evaluate the importance of the mimicry phenomenon in the use of smartphones at a large scale, it would be interesting to check if the visual static/dynamic advertisements involving users looking at their devices are more effective than those not showing any user but only the device.

Finally, it is difficult to say whether the mimicry response we recorded in the use of smartphones was affected by the lockdown imposed by the Italian Government from March 9th to May 18th 2020 due to the COVID-19 pandemic. We do not know if our findings are linked to the previous period of forced social isolation during which people relied almost entirely on their devices to keep in contact with others and maintain their social bonding. A long-term data collection on mimicry in the use of smartphones will be necessary to explore the possible effect of the lockdown on this intriguing phenomenon.

## Supplementary Information

Below is the link to the electronic supplementary material.

Supplementary file1 (CSV 18 KB)

Supplementary file2 (CSV 6 KB)
